# L-carnitine: new perspectives on the management of preterm infants

**DOI:** 10.3389/fnut.2025.1508441

**Published:** 2025-08-29

**Authors:** Mo Sisi, Lin Yong, Qiao Lixing, Guo Changsheng, Yao Jin, Zhou Hang, Cao Xing, Liu Heng

**Affiliations:** ^1^Department of Pediatrics, Zhongda Hospital, Southeast University, Nanjing, China; ^2^Department of Pediatrics, Affiliated Taikang Xianlin Drum Tower Hospital, Medical School of Nanjing University, Nanjing, China; ^3^Department of Respiratory, Children’s Hospital of Nanjing Medical University, Nanjing, China

**Keywords:** L-carnitine, preterm infants, neurodevelopment, hypoxic–ischemic encephalopathy, respiratory distress syndrome

## Abstract

L-carnitine, a quaternary ammonium compound derived from amino acids, serves an essential role in fatty acid metabolism. The functions of L-carnitine include assisting long-chain fatty acyl-CoA across the mitochondrial membrane to promote mitochondrial *β*-oxidation, reducing oxidative stress damage, and maintaining cellular energy homeostasis. Therefore, postnatal L-carnitine deficiency may lead to impaired fatty acid oxidation, resulting in clinical manifestations of hypoglycemia, hypothermia, acidosis and infection. However, there is still no clear consensus on the need for prophylactic use of L-carnitine in the treatment of preterm infants. This review synthesizes the theoretical foundations and clinical evidence for L-carnitine in preterm infant management, revealing that L-carnitine exerts demonstrable effects on promoting neurodevelopment and preventing neonatal complications. Furthermore, it explores the potential value and current controversies surrounding its prophylactic application.

## 1 Introduction

As a conditionally essential nutrient, L-carnitine has an important role in human fat metabolism. Moreover, L-carnitine can synthesize acetyl-L-carnitine in the body, which has neuro-trophic and neuro-protective effects (1). Due to its good tolerance and few side effects, L-carnitine has been approved as a dietary supplement in formula milk or as a drug in some countries (2).

Premature infants are prone to L-carnitine deficiency (3, 4). This is related to factors such as insufficient gestation time, which prevents the placenta from accumulating and storing sufficient amounts of L-carnitine, lower oral food intake after birth, and a high metabolic rate in newborns (5). L-carnitine supplementation by intravenous infusion can improve the level of L-carnitine in premature infants who cannot be fully fed by mouth (6, 7). By adjusting lipid energy use efficiency and the metabolite profile, the tolerance of children to unstable factors is improved. However, whether L-carnitine supplementation is necessary for premature infants remains controversial. This review synthesizes current knowledge with regard to biological roles, metabolic profiles of L-carnitine in preterm infants, effects of supplementation on complications, and existing controversies.

## 2 Biological function of L-carnitine

**Figure 1:**
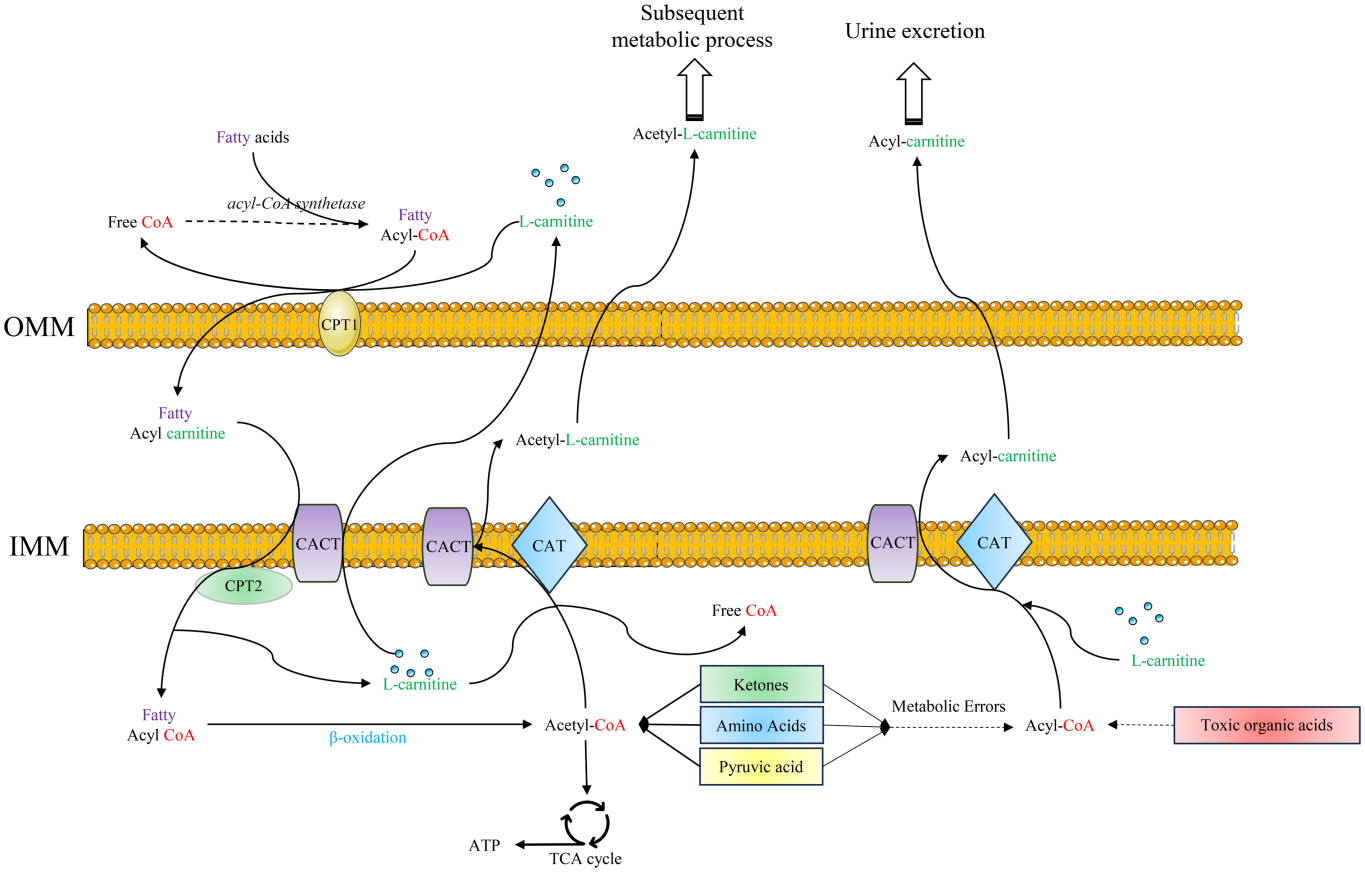
Functional mechanisms of L-carnitine: ① Fatty acids are activated to fatty acyl-CoA, which is shuttled into the mitochondria facilitated by L-carnitine, CPT1, CPT2, and the CACT. Subsequently, fatty acyl-CoA undergoes β-oxidation to generate acetyl-CoA. This acetyl-CoA then enters the TCA cycle, yielding ATP for energy production; ② Within the mitochondria, L-carnitine facilitates the action of CACT and CAT to convert acetyl-CoA to acetylcarnitine. This process regulates the acetyl-CoA/CoA ratio, thereby modulating the metabolism of various substrates. The generated acetyl-L-carnitine is transported to the cytoplasm to participate in other metabolic pathways; ③ Acyl-CoAs derived from the abnormal metabolism of ketone bodies, amino acids, and pyruvate, as well as from toxic organic acids, are conjugated to L-carnitine. Facilitated by CACT and CAT, these acyl groups are converted to acyl-carnitines, which are transported across the mitochondrial membrane and ultimately excreted renally. OMM: outer mitochondrial membrane; IMM: inner mitochondrial membrane; CPT1: carnitine palmitoyl transferase 1; CPT2: carnitine palmitoyl transferase 2; CACT: carnitine acylcarnitine translocase; CAT: carnitine transferase; TCA cycle: tricarboxylic acid cycle.

L-carnitine works together with carnitine palmitoyl transferase (CPT) and carnitine-acylcarnitine translocase (CACT) on the mitochondrial membrane to form an efficient transport system (8). In this transport system, CPT and CACT can regulate the transport mode according to the changes of metabolites in mitochondria, playing an important role in the transport and metabolism of fat, carbohydrates, proteins, and toxic substances produced by abnormal metabolism (Figure 1) (9, 10).

### 2.1 Fatty acid metabolism

L-carnitine promotes fatty acid oxidation for energy. The function of fat oxidation occurs mainly in mitochondria to ultimately release energy for the body to use. During this process, fat must traverse the mitochondrial membrane. However, long-chain fatty acids are incapable of permeating this barrier and gaining entry into the mitochondria for *β*-oxidation. Firstly, long-chain fatty acids are converted to long-chain acyl-CoA catalyzed by acyl-CoA synthetases in the endoplasmic reticulum and outer mitochondrial membrane. Subsequently, CPT1 catalyzes the association of long-chain fatty acyl-CoA with L-carnitine to generate acylcarnitine. Following this, acylcarnitine is transferred to the mitochondrial matrix by CACT, which is located in the inner mitochondrial membrane. Finally, in the mitochondrial matrix, acylcarnitine is resolved by CPT2 to acyl-CoA and L-carnitine. Fatty acyl-CoA undergoes *β*-oxidation within mitochondria to produce energy, and L-carnitine is transported to the cytoplasm again by CACT (As show in Figure 1). Fat emulsion, which contains medium- and long-chain fatty acids, plays an important role in parenteral nutrition in premature infants (11). Studies have shown that supplementation of L-carnitine in parenteral nutrition can significantly improve fatty acid metabolism in neonates (12, 13).

### 2.2 Improving metabolic flexibility

Mitochondria not only represent a site for fat *β*-oxidation but are also a reaction site for metabolic processes such as protein synthesis/breakdown (14). The relatively high concentration of free CoA in mitochondria help to maintain the metabolic diversity of substances. CoA participates in the synthesis of a large number of essential substances; for example, synthesis of the neuromuscular messenger and neurotransmitter acetylcholine (15, 16). L-carnitine is a carrier of active acetyl and acyl groups (17). In partnership with CPT and CACT, L-carnitine forms an effective transport system for acetyl groups or acyl groups. L-carnitine plays a key role in regulating acyl-CoA and acetyl-CoA and in maintaining free CoA levels within mitochondria, which is essential for maintaining metabolic flexibility (Figure 1) (18).

### 2.3 Detoxification of potentially toxic metabolites

A critical function of L-carnitine involves the detoxification of potentially toxic metabolites generated during intermediary metabolism, particularly those associated with impaired fatty acid *β*-oxidation. L-carnitine accomplishes this by binding to acyl residues produced not only from fatty acids but also from amino acid metabolism, forming water-soluble acylcarnitines that facilitate their excretion from cells and ultimately the body (Figure 1) (19, 20). This detoxification mechanism is especially vital in disorders of mitochondrial fatty acid oxidation (FAO). The relevance of this detoxification pathway extends significantly to the vulnerable preterm infant population. Preterm infants frequently encounter metabolic stressors such as hypoxia and infection. These stressors can disrupt normal metabolic flux, potentially leading to the accumulation of toxic intermediates within cells and altering cellular metabolic homeostasis (21). Compounding this vulnerability, the immature gastrointestinal tract of preterm infants faces specific risks. Animal models mimicking preterm physiology have demonstrated that exposure of the intestinal mucosa to certain fatty acid derivatives can induce mucosal damage, including necrosis (22). Mechanistically, because acylcarnitines are direct metabolites derived from fatty acid and organic acid catabolism, disruptions in systemic FAO under metabolic stress, such as during the introduction of enteral feeding in a compromised gut, could theoretically contribute to gut-specific toxicity. The accumulation of potentially toxic acyl species or imbalances in acylcarnitine profiles within the immature intestine might play a role in such injury pathways.

### 2.4 Stabilization of cell membranes

L-carnitine is also essential in maintaining membrane stability and the function of plasma, mitochondria, and other organelles, probably through its effects on the acetylation of membrane phospholipids. The amphiphilicity of L-carnitine also permits interaction with surface charges on the cell membrane and may play a role in membrane stabilization. The charged trimethylamine and carboxyl groups on L-carnitine facilitate interaction with the corresponding poles on membrane phospholipids, glycolipids, and proteins (23, 24).

### 2.5 Control of ketogenesis

**Figure 2:**
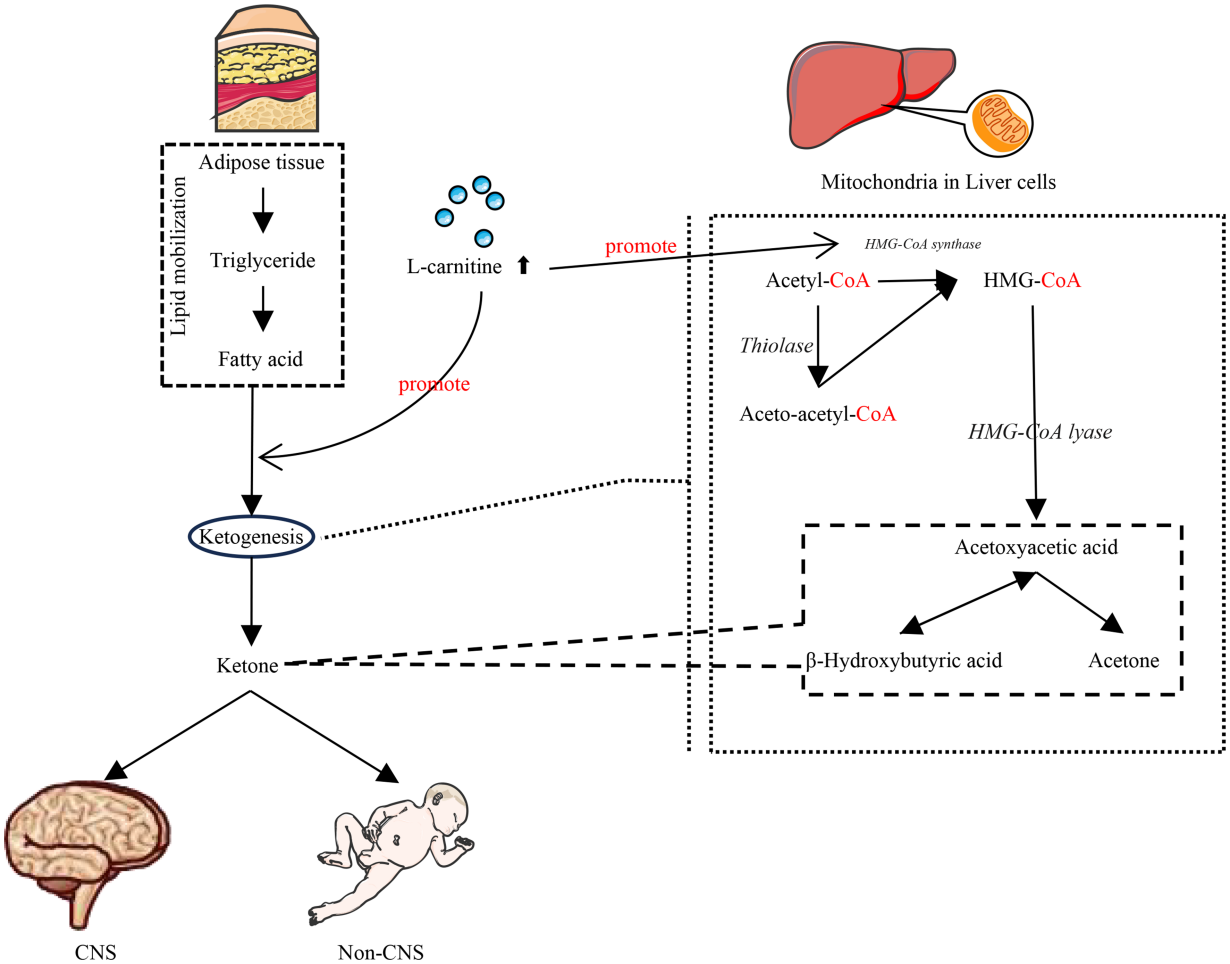
L-Carnitine facilitates ketogenesis. L-carnitine supplementation enhances mitochondrial fatty acid transport and modulates key enzyme activities and gene expression to facilitate ketogenesis, thereby ensuring central nervous system function and fundamental physiological demands during glucose insufficiency.

L-carnitine plays an indispensable role in this ketogenic pathway. Ketogenesis occurs predominantly within the hepatic mitochondria, with carnitine palmitoyl transferase I (CPT-1) acting as the critical rate-limiting enzyme for mitochondrial fatty acid import (Figure 1). Fatty acids that enter the mitochondria via CPT-1 are broken down into acetyl-CoA by *β*-oxidation. Two acetyl-CoA molecules are converted to acetoacetic-CoA, then to hydroxy methylglutaryl coenzyme A (HMG-CoA), and subsequently to acetoacetate. Once acetoacetate reaches extrahepatic tissues, it is converted back to acetyl-CoA, which can enter the citric acid cycle to produce ATP. It is essential for the initial transport of activated long-chain fatty acids across the mitochondrial membrane via the CPT system, thus governing the substrate flux available for both *β*-oxidation and subsequent ketogenesis. Consequently, the efficiency of ketone body production is critically dependent on adequate L-carnitine availability (Figure 2). L-carnitine deficiency severely impairs the mitochondrial import of long-chain acyl-CoAs, directly limiting the acetyl-CoA pool required for ketogenesis (3). L-carnitine facilitates ketogenesis by modulating key enzyme activities and gene expression. Specifically, it up-regulates CPT1 mRNA expression, thereby promoting mitochondrial fatty acid transport and enhancing *β*-oxidation activity (25, 26). Concurrently, the expression of HMG-CoA synthase—a pivotal ketogenic enzyme—may undergo analogous modulation, while HMG-CoA reductase activity (its downstream counterpart) is suppressed, thereby reducing fatty acid conversion to cholesterol. Furthermore, this process inhibits the expression of lipogenesis-related genes, ultimately enhancing ketogenesis (27, 28).

The preterm brain exhibits extraordinarily high energy demands to fuel rapid growth and development, demands which can surpass the capacity of glucose supply alone. Under these circumstances, ketone bodies—especially *β*-hydroxybutyrate derived from FAO—serve as vital alternative energy substrates (29, 30). Beyond mere fuel provision, ketone bodies actively support critical neurodevelopmental processes, including neural stem cell proliferation, differentiation, and functional maintenance. This dependency on keton is particularly crucial for the developing brain of preterm infants. Notably, neonatal ketogenesis possesses unique characteristics: it can be readily activated even in the non-fasting state, providing essential protective effects during the metabolically vulnerable perinatal period. These protective effects are thought to stem from maintaining cerebral energy homeostasis and mitigating metabolic stress (31). Given the pivotal role of L-carnitine in enabling ketogenesis and the heightened reliance of the preterm brain on ketone bodies, strategic L-carnitine supplementation represents a potential therapeutic approach. The goal would be to safely elevate and sustain ketone body levels in preterm infants, thereby optimizing cerebral energy metabolism while carefully managing potential metabolic risks (32).

## 3 Metabolic characteristics of L-carnitine in preterm infants

### 3.1 L-carnitine sources

There are three main sources of L-carnitine in newborns: endogenous synthesis, pre-natal storage, and post-natal exogenous supplementation. Carnitine synthesis requires gamma-butyl betaine hydroxylase, and premature infants have a limited capacity to synthesize L-carnitine themselves, having only 12–15% of the synthesis ability of adults (33–35). Therefore, newborns are more dependent on the latter two sources of L-carnitine (34).

### 3.2 L-carnitine levels in preterm infants

Due to the large transfer of maternal L-carnitine to the fetus in the third trimester and the immature mechanism of carnitine transport in premature infants, the blood level of l-carnitine in premature infants after 3 to 7 days is slightly higher than that in full-term infants (5). When monitoring the blood carnitine level in premature and term infants, it was found that the carnitine level gradually increased after birth, and the L-carnitine level could increase by one-third at 1 month after birth compared with that after birth. However, in premature infants, especially very premature infants requiring long-term parenteral nutrition, L-carnitine levels continued to decrease within 1 month after birth (36, 37). The fetus accumulates substantial amounts of L-carnitine, primarily during the third trimester, through transplacental transfer from the mother, storing it predominantly in muscle tissue. While preterm infants are born before completing this critical accretion phase, resulting in lower total body L-carnitine stores compared to term infants, their immediate postnatal peripheral blood concentrations may paradoxically be elevated. This transient elevation is attributable to the immaturity of carnitine transport and utilization mechanisms in preterm infants (38, 39).

Consequently, despite the potential for higher initial blood levels, the inherent physiological immaturity and reduced somatic mass associated with preterm birth mean that these infants have significantly diminished endogenous L-carnitine reserves. Furthermore, under normal physiological conditions following birth, term newborns readily acquire sufficient L-carnitine through enteral feeds. However, achieving adequate enteral intake can be delayed or compromised in preterm infants, particularly those who are very immature or critically ill, heightening their vulnerability to carnitine insufficiency (40).

## 4 The impact of supplementation of L-carnitine on preterm infants

### 4.1 Effect of L-carnitine supplementation on L-carnitine levels in preterm infants

**Figure 3:**
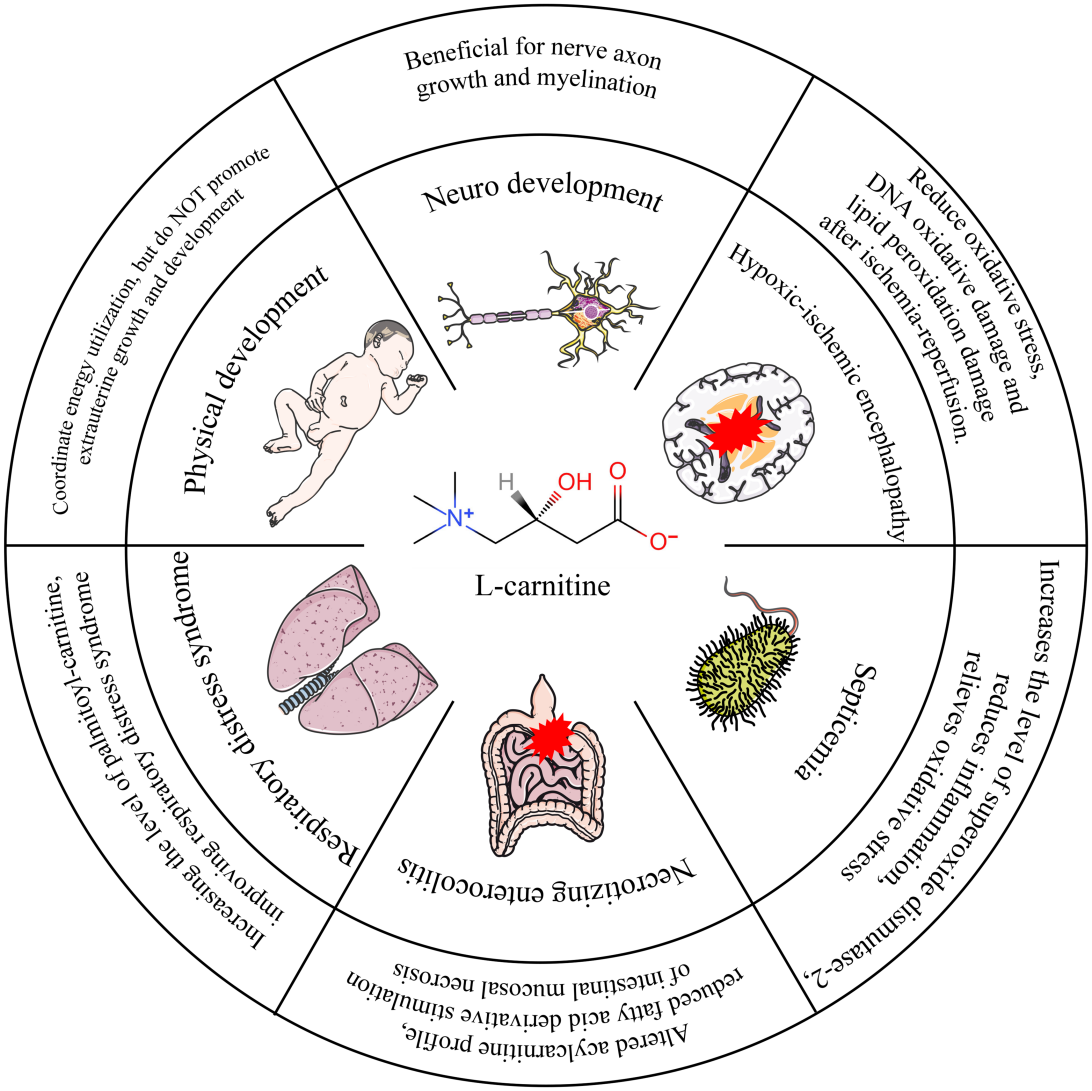
Overview of functional L-carnitine in different diseases during preterm infants. ① L-Carnitine may confer transient benefits on early weight gain in specific preterm infant subpopulations. From a comprehensive assessment, however, it demonstrates no positive impact on overall physical growth and development. ② L-Carnitine promotes volumetric growth of cerebral tissue in preterm infants. ③ Neuroprotective effects of L-Carnitine are evidenced in both adult patients with hypoxic–ischemic encephalopathy and corresponding animal models. ④ Supplementation reduces levels of inflammatory markers and oxidative stress, thereby ameliorating clinical manifestations in septic patients. ⑤ By normalizing aberrant acylcarnitine metabolic profiles through supplementation, progression to NEC may be mitigated. ⑥ L-Carnitine supplementation diminishes the requirement for exogenous pulmonary surfactant in infants with RDS.

L-carnitine supplementation with parenteral nutrition can significantly increase the L-carnitine concentration in preterm infants (41). In one study, 29 preterm infants with gestational age < 32 weeks, weight < 1,500 g, and age < 4 days after birth were randomly divided into two groups and supplemented with L-carnitine 20 mg/(kg per day) and placebo to detect the intracellular left-carnitine level in premature infants and preterm infants. The authors found that the mean level of L-carnitine was higher in preterm infants, and the number of cases with normal L-carnitine levels in red blood cells after supplementation was significantly higher than that in the placebo group (42). In another study, the researchers looked at parenteral nutrition in 982 premature infants weighing < 1,500 g at 23 neonatal treatment centers in the United States. The results showed that 390 children who received L-carnitine supplementation had higher mean levels of L-carnitine than children without L-carnitine supplementation (40). Evidences suggests that supplementing L-carnitine in parenteral nutrition (PN) regimens can significantly enhance fatty acid metabolism in neonates (12, 13). Within the components of PN, intravenous fat emulsions—providing vital medium- and long-chain fatty acids—play an indispensable role in meeting the high energy demands and supporting the development of premature infants (11). Crucially, fatty acids serve as a primary energy substrate and are essential for critical processes such as brain development and cell membrane synthesis. Notably, efficient fatty acid metabolism, particularly beta-oxidation for energy production, requires adequate levels of L-carnitine, which acts as an essential cofactor for transporting long-chain fatty acids into the mitochondria. This biochemical rationale provides a strong theoretical basis for L-carnitine supplementation in PN (Figure 3).

### 4.2 Effect on extrauterine growth restriction

Supplementation with L-carnitine lacks robust evidence for improving extrauterine growth. While early studies demonstrated that L-carnitine supplementation could promote physical growth in preterm infants, primarily evidenced by enhanced weight gain, current evidence supporting its efficacy in ameliorating extrauterine growth restriction remains insufficient. Earlier studies reported associations between PN carnitine supplementation and improved physical development outcomes in preterm infants (43, 44). However, Whitfield et al. conducted a randomized trial in 80 preterm infants weighing < 1,500 g, supplementing either L-carnitine (15 mg/kg per day) or placebo. They meticulously monitored length, weight, head circumference, and subcutaneous fat thickness weekly until an adjusted gestational age of 36 weeks, yet found no significant differences in any of these physical development indicators between the groups (45). Similarly, Pande et al. reported no significant effect of PN L-carnitine supplementation on physical development indicators in their cohort of preterm infants (34). A more nuanced finding comes from Crill et al., who observed that preterm infants receiving PN with L-carnitine supplementation achieved weight gain 5 days earlier than the placebo group; nevertheless, no statistically significant difference in weight was observed at the 8-week endpoint between the two groups (42). It is important to note that a key methodological heterogeneity across these studies is the variation in L-carnitine supplementation doses (e.g., 15 mg/kg/day in Whitfield et al. vs. 20 mg/kg/day in some earlier positive studies). This lack of dose standardization complicates direct comparisons and may contribute to the discrepancies in reported outcomes regarding physical development. Furthermore, factors such as study population characteristics, duration of supplementation, and the specific growth parameters measured (short-term weight gain velocity vs. longer-term auxological parameters) likely influence the observed effects.

Based on the available data, intravenous L-carnitine supplementation may offer a transient benefit for early weight gain in certain preterm infant populations. From a comprehensive perspective, L-carnitine does not exhibit a positive impact on the promotion of physical growth and development.

### 4.3 Effect on neuro-developmental

Throughout the developmental process, the brain exhibits heightened energy demands for synthesizing fundamental cellular structures, neurotransmitters, nucleic acids, proteins, carbohydrates, and lipids that are indispensable for neuronal growth and myelination (46, 47). Critically, adequate energy substrate utilization is essential for achieving optimal brain growth and maturation. Consequently, cerebral tissue volume and white matter microstructure, both energy-dependent processes, are key determinants of neurological developmental outcomes (47–49). L-carnitine plays a pivotal role in meeting the brain’s substantial energy requirements. It acts as an essential mitochondrial membrane transporter for long-chain fatty acids, facilitating their *β*-oxidation—a major energy-yielding pathway. Significantly, L-carnitine can traverse the blood–brain barrier, positioning it to directly support cerebral energy metabolism and function (50). Supporting this mechanistic role, observational studies indicate that initial carnitine uptake and serum L-carnitine levels in preterm infants during the early postnatal weeks are independently associated with brain size. These findings suggest that free and short-chain acyl-carnitines may exert distinct effects on brain growth trajectories (51, 52). The importance of L-carnitine availability for brain development is further underscored by nutritional studies. Breastfeeding, which provides a rich source of L-carnitine among other bioactive factors, has been associated with promoting regional brain volume and cerebellar development in preterm infants (3, 51, 53). More directly, such supplementation has also been shown to augment cerebral parenchymal volume at term-equivalent age in extremely preterm infants (3).

In summary, converging evidence from mechanistic, observational, and interventional research suggests that L-carnitine availability likely contributes to brain tissue growth in preterm infants. While the data point to beneficial effects on structural brain development, further investigation is crucial to determine the long-term impact of L-carnitine status and supplementation on functional neuro-developmental outcomes, such as cognitive and motor abilities, in this vulnerable population.

### 4.4 Neonatal hypoxic–ischemic encephalopathy

The pathophysiology of HIE involves hypoxic–ischemic injury, characterized by perturbations in mitochondrial dynamics and the inhibition of critical energy-producing pathways, including oxidative phosphorylation and fatty acid *β*-oxidation (54). L-carnitine has demonstrated efficacy as a neuroprotective agent in adult animal models of cerebral ischemia (55). Currently, the cornerstone therapy for moderate to severe neonatal HIE is therapeutic hypothermia. While hypothermia provides significant neuroprotection, its proposed mechanisms are multifactorial and may include modulation of neurotransmitter systems and potentially influencing metabolic pathways involving compounds like carnitine (56). Nevertheless, therapeutic hypothermia is only partially effective (57–60), and many infants with HIE remain at substantial risk of death or severe long-term neurodevelopmental impairments (55). This significant unmet clinical need underscores the urgency for developing adjunctive or alternative neuroprotective strategies. Given L-carnitine established role in facilitating mitochondrial *β*-oxidation and its prior neuroprotective effects in adult ischemia models, it represents a biologically plausible candidate for investigation in neonatal brain injury.

### 4.5 Septicemia

Bacterial inflammation triggers significant oxidative stress, during which the metabolism of oxidized phospholipids is regulated, at least in part, by mitochondrial β-oxidation pathways (61). This oxidative milieu can damage cellular components, including enzymes critical for energy metabolism. Therefore, therapeutic agents capable of mitigating oxidative damage are of significant interest. L-carnitine has emerged as one such candidate, with evidence suggesting it acts protectively by scavenging free radicals and enhancing the activity of endogenous antioxidant enzymes, thereby shielding vital enzymes from oxidative inactivation (62). Supporting this mechanism, studies in animal models demonstrate that L-carnitine administration increases the expression of mitochondrial superoxide dismutase-2 (SOD2), reduces markers of inflammation, and alleviates overall oxidative burden (63). Translating these preclinical findings to the clinical setting, a randomized, double-blind, controlled trial investigated the impact of L-carnitine supplementation in critically ill adults with sepsis. This study reported that L-carnitine supplementation effectively reduced levels of inflammatory markers and oxidative stress, and importantly, was associated with improved clinical outcomes (64). While this evidence in adults is promising, further research is warranted to specifically evaluate the potential role and efficacy of L-carnitine in modulating inflammation and oxidative stress related to bacterial infections in pediatric populations, particularly where mitochondrial dysfunction may play a key role.

### 4.6 Effect on neonatal necrotizing enterocolitis

The protective effects of levocarnitine supplementation against hypoxia/re-oxygenic necrotizing enterocolitis (NEC) have been demonstrated in young mice (65). Premature neonates are susceptible to NEC due to impaired fatty acid metabolism. Because acyl-carnitine is derived from the metabolism of fatty acids and organic acids, it is reasonable that abnormal systemic fatty acid oxidation readily leads to gut-specific toxicity after the introduction of a metabolic challenge with enteral feeding. Animal models of preterm infants have reported that exposure of the intestinal mucosa to fatty acid derivatives causes mucosal necrosis (66, 67). In another study, researchers found that abnormal acylcarnitine metabolic profiles existed before the occurrence of NEC. With L-carnitine supplementation, we can improve the blood L-carnitine level and improve the acylcarnitine profile, thereby avoiding the development of NEC (68).

### 4.7 Effect on neonatal respiratory distress syndrome

Premature infants diagnosed with neonatal respiratory distress syndrome (RDS) exhibit significantly diminished plasma levels of L-carnitine compared to their non-RDS counterparts (69). Critically, this disparity persists under comparable nutritional support regimens, with plasma free carnitine concentrations remaining significantly lower in RDS infants from day 3 to day 7 postpartum (69). This sustained deficiency likely reflects a substantial increase in L-carnitine utilization during the critical first postnatal week in preterm infants with RDS (70). The potential clinical significance of L-carnitine status in RDS extends beyond energy metabolism. L-carnitine serves as a precursor for palmitoyl-carnitine, a significant constituent of pulmonary surfactant—the essential lipoprotein complex crucial for maintaining alveolar stability and preventing atelectasis (71, 72). Given pulmonary surfactant’s pivotal role in RDS pathophysiology and the dependency of palmitoyl-carnitine synthesis on adequate L-carnitine availability, strategies to augment L-carnitine levels represent a biologically plausible approach to support surfactant production and potentially ameliorate RDS severity.

Emerging clinical evidence lends support to this concept across different intervention timepoints: ① Prenatal/Perinatal Strategy: A randomized study involving 100 pregnant women at high risk for preterm delivery compared prophylactic administration of L-carnitine plus dexamethasone versus dexamethasone alone. The findings demonstrated that infants born to mothers receiving the combination therapy had a significantly reduced incidence of RDS and lower mortality rates compared to the dexamethasone-only group (70). ② Postnatal Strategy: A randomized controlled trial enrolled preterm infants (28–36 weeks gestation) diagnosed with RDS within 6 h of birth. Infants received either L-carnitine supplementation (30 mg/kg/day) or placebo via parenteral nutrition during the first postnatal week. Consistent with the proposed mechanism, infants supplemented with L-carnitine exhibited significantly elevated plasma carnitine levels by day 7. Furthermore, they demonstrated clinically relevant benefits, including a reduced requirement for exogenous pulmonary surfactant (both in the number of infants needing treatment and the total dose administered) and a shortened duration of mechanical ventilation compared to the placebo group (73).

While these preliminary studies—demonstrating benefits from both prenatal/perinatal and postnatal L-carnitine supplementation—are promising and mechanistically coherent, it is imperative to acknowledge the current limitations. Specifically, robust evidence from large-scale, multicenter randomized controlled trials is still needed to conclusively establish efficacy, determine optimal dosing and timing strategies, and fully evaluate safety before widespread clinical adoption in RDS management can be recommended. Nevertheless, the existing mechanistic rationale coupled with these encouraging early clinical observations positions L-carnitine as a compelling candidate for further investigation as a potential adjunctive therapy for neonatal RDS.

### 4.8 Other applications of L-carnitine in pediatrics

Beyond its roles in energy metabolism and neuroprotection, L-carnitine supplementation demonstrates potential benefits in other pathological contexts relevant to pediatrics. Animal studies and clinical trials suggest that L-carnitine can improve nitrogen balance under pathological stress by enhancing protein synthesis, reducing protein breakdown, inhibiting apoptosis, and mitigating inflammatory responses (74). Furthermore, L-carnitine positively influences key pathways implicated in pathological skeletal muscle wasting. This modulation may underlie at least some of the observed anti-catabolic effects and improvements in fatigue-related outcomes reported in chronic disease patients receiving L-carnitine (75). Consequently, severe L-carnitine deficiency itself can precipitate syndromes characterized by energy metabolism dysfunction.

Shifting focus to a critical neonatal complication, hyperoxia-induced lung injury is a significant concern in preterm infants. Hyperoxia exposure impairs mitochondrial respiratory capacity in premature lung endothelial cells, ultimately triggering apoptosis. This cellular damage contributes to clinical sequelae such as prolonged mechanical ventilation dependence and surfactant deficiency (76). Mechanistically, studies indicate that the transition from hyperoxia back to normoxia paradoxically suppresses FAO while promoting ceramide synthesis, both pathways converging to promote apoptosis. Crucially, experimental interventions targeting FAO modulate this injury: Enhancing FAO with L-carnitine reduced hyperoxia-induced impairments in alveolarization and vascularization, whereas inhibiting FAO exacerbated these injuries. Thus, in neonatal models, hyperoxia exerts long-term detrimental effects on lung development partly through dysregulated FAO, and therapeutic strategies like L-carnitine supplementation that augment FAO show promise in mitigating hyperoxia-induced lung damage (77).

Apnea of prematurity represents another frequent challenge in this vulnerable population. Evidence regarding the impact of parenteral L-carnitine supplementation on apnea incidence is currently mixed. One study found it reduced apnea in very preterm infants (42). However, a randomized controlled trial by Donnell et al. involving preterm infants < 32 weeks gestation, weighing < 1,500 g, and enrolled < 4 days of age, reported no significant difference in apnea frequency between L-carnitine supplemented infants and controls (78). This latter study also concluded that supplementation did not alter apnea occurrence in infants < 1,500 g. It is noteworthy that methodological limitations, such as the absence of nasal thermistor monitoring in the Donnell et al. study, may have compromised apnea detection, particularly for obstructive and mixed events, potentially leading to underreporting (79). The discrepancies observed across studies likely stem from variations in patient selection criteria, L-carnitine dosing regimens, and importantly, the sensitivity and methodology of apnea monitoring. Therefore, while preliminary findings exist, robust evidence confirming a beneficial effect of parenteral L-carnitine supplementation on apnea of prematurity remains limited. Further well-designed, multicenter randomized controlled trials employing standardized and sensitive apnea detection methods are essential to definitively evaluate this potential application (45, 78).

## 5 Discussion

The survival rates of preterm infants in developed and developing countries continue to improve, which may reflect improvements in nutrition and health care. However, preterm infants still have a high risk of developing complications and poor outcomes, and this risk is even higher for infants born below their gestational age. Therefore, numerous studies have focused on improving the nutritional status of preterm infants, reducing the incidence of complications, and improving long-term outcomes.

Preterm infants are not efficient in their energy use, a poor exogenous nutrient supply during the first few days of life may not only lead to extrauterine growth retardation in newborns, but it may also increase their susceptibility to infectious diseases and organ function damage (80). L-carnitine plays an important role in maintaining metabolic flexibility in the human body. The most important function of L-carnitine is to obtain cellular energy from fatty acids in the mitochondrial matrix. L-carnitine also maintains the stability of mitochondrial CoA in the process of fatty acid oxidation, thereby maintaining metabolic flexibility (81). Another function of carnitine in human metabolism is its involvement in the detoxification process of toxic compounds (such as some foreign substances, including ampicillin, valproic acid, and salicylic acid, as well as toxic substances generated endogenously via metabolism), which are jointly excreted by the kidneys with carnitine (82). Additionally, L-carnitine exhibits antioxidant and membrane stabilizing effects by improving biological electrical activity and inhibiting the production of free radicals. Finally, based on changes in energy supply sources, L-carnitine regulates the utilization of glucose synthesis and fatty acid oxidation, providing the organism with a certain buffering capacity to cope with complex changes.

Despite the positive role of L-carnitine in regulating metabolism, prophylactic supplementation of L-carnitine in preterm infants does not seem to achieve the expected results. Studies have found that supplementing L-carnitine through parenteral nutrition can improve the acylcarnitine profile in the peripheral blood of preterm infants. However, evidences suggest that L-carnitine does not have a positive effect on the physical development of preterm infants (34, 42, 45). This may be related to the rich experience of evidence-based medicine in the treatment of preterm infants and optimization of the energy ratios in intravenous nutrition show that the role of L-carnitine in promoting the physical development of preterm infants seems to be irrelevant when the calorie supply is sufficient. However, L-carnitine plays a positive role in the neurodevelopment of preterm infants. Currently available research suggests that consuming a moderate amount of L-carnitine may help increase its serum levels, and serum concentrations of L-carnitine are positively correlated with extremely preterm infant brain volume growth. Brain capacity is not the only determinant of brain function (3). However, the current research is limited with regard to exploring the effects of L-carnitine on brain function. This necessitates high-quality cohort studies or randomized controlled trials (RCTs) to evaluate the efficacy of L-carnitine in enhancing neuro-functional outcomes.

Including evidence from observational study, other evidence supports the view that L-carnitine may be beneficial for the prevention and treatment of complications in preterm infants. For example, reducing the use of pulmonary surfactant in children with neonatal respiratory distress syndrome (73), reducing mechanical ventilation time (70) are all potential therapeutic effects of L-carnitine against complications in preterm infants. Further clarification is needed on the appropriate supplemental dose and corresponding time points. Studies have shown that adult patients with hypoxic–ischemic injury can improve their cognitive function after supplementation with L-carnitine (83). In animal models of neonatal hypoxic–ischemic encephalopathy, the same positive results were obtained (84, 85). Critically, however, direct evidence evaluating the efficacy of L-carnitine specifically for treating neonatal cerebral ischemic injury is currently lacking. Therefore, well-designed *in vivo* studies, incorporating long-term neuro-developmental follow-up, are essential to rigorously evaluate the therapeutic potential of L-carnitine/acetyl-L-carnitine in protecting the vulnerable developing brain following HIE (86).

In addition, the theoretical effect of L-carnitine is not limited to the above mentioned clinical applications. Cell and animal experiments have also suggested that L-carnitine is beneficial for reducing the occurrence of complications of preterm birth, such as NEC, septicemia, and improving the short- and long-term prognosis in. However, these studies are still at the level of mechanism and animal experiments. There is currently no conclusive clinical research evidence to support this. Therefore, exploring the potential of L-carnitine in improving complications among preterm infants and their corresponding short- and long-term outcomes may provide new interesting research opportunities toward further optimizing the treatment of preterm infants.

## 6 Conclusion

The impact of poor outcomes in preterm infants has prompted the exploration and optimization of new treatment regimens. In our study, we reviewed the theoretical basis and clinical evidence for the use of L-carnitine in the treatment of preterm infants. There appears to be no clearly positive effect of L-carnitine on preterm infant physical development, the published evidence shows potential for promoting neurodevelopment, reducing the incidence of comorbidities, and improving the prognosis of preterm infants. Current evidence indicates a reassuring safety profile of L-carnitine in preterm infants, with no adverse effects observed across studies despite substantial variations in dosing regimens (41), but we remain recommend further research to establish the optimal dosing regimen. Then conduct adequately powered cohort studies or RCTs to definitively determine its efficacy in improving neurodevelopmental outcomes and preventing complications. Future research should include appropriately powered cohort studies or randomized controlled trials (RCTs) to: ① Investigate the prophylactic efficacy of L-carnitine against preterm infant complications [e.g., sepsis, necrotizing enterocolitis (NEC)]; ② Establish optimal dosing regimens and treatment duration for respiratory distress syndrome (RDS) management; ③ Assess neurodevelopmental promotion and neurological repair through longitudinal follow-up studies. These evidences are crucial for optimizing current therapeutic strategies for preterm infants and ultimately enhancing their quality of life.

## References

[1] Da Silva LE de Oliveira MP da Silva MR Abel JS Tartari G de Aguiar da Costa M. L-carnitine and acetyl-L carnitine: a possibility for treating Alterat ions induced by obesity in the central nervous system. Neurochem Res. (2023) 48:3316–26. doi: 10.1007/s11064-023-04000-z, PMID: 37495838

[2] Bakshi S Paswan VK Yadav SP Bhinchhar BK Kharkwal S Rose H. A comprehensive review on infant formula: nutritional and functional constituents, recent trends in processing and its impact on infants’ gut microbiota. Front Nutr. (2023) 10:1194679. doi: 10.3389/fnut.2023.1194679, PMID: 37415910 PMC10320619

[3] Manninen S Silvennoinen S Bendel P Lankinen M Schwab US Sankilampi U. Carnitine intake and serum levels associate positively with postnatal growth and brain size at term in very preterm infants. Nutrients. (2022) 14:725. doi: 10.3390/nu14224725, PMID: 36432412 PMC9696952

[4] Wang L Zhong WH Liu DY Shen HQ He ZJ. Metabolic analysis of infants with bronchopulmonary dysplasia under early nutrition therapy: an observational cohort study. Exp Biol Med (Maywood). (2022) 247:470–9. doi: 10.1177/15353702211060513, PMID: 34894806 PMC8943329

[5] Qi ZY Duan J Wang Q Yao Q Zhong QH Zhang CY. Levels of blood free carnitine in preterm infants with different gestational ages and birth weights. Zhongguo Dang Dai Er Ke Za Zhi. (2019) 21:562–6. doi: 10.7499/j.issn.1008-8830.2019.06.012, PMID: 31208510 PMC7389571

[6] Seong SH Cho SC Park Y Cha YS. L-carnitine-supplemented parenteral nutrition improves fat metabolism but fails to support compensatory growth in premature Korean infants. Nutr Res. (2010) 30:233–9. doi: 10.1016/j.nutres.2010.04.004, PMID: 20534325

[7] Fohner AE Garrison NA Austin MA Burke W. Carnitine palmitoyltransferase 1A P479L and infant death: policy implications of emerging data. Genet Med. (2017) 19:851–7. doi: 10.1038/gim.2016.202, PMID: 28125087 PMC5620101

[8] Gnoni A Longo S Gnoni GV Giudetti AM. Carnitine in human muscle bioenergetics: can carnitine supplementation improve physical exercise? Molecules. (2020) 25:182. doi: 10.3390/molecules25010182, PMID: 31906370 PMC6982879

[9] Virmani A Pinto L Bauermann O Zerelli S Diedenhofen A Binienda ZK. The carnitine Palmitoyl transferase (CPT) system and possible relevance for neuropsychiatric and neurological conditions. Mol Neurobiol. (2015) 52:826–36. doi: 10.1007/s12035-015-9238-7, PMID: 26041663

[10] Wanders RJA Visser G Ferdinandusse S Vaz FM Houtkooper RH. Mitochondrial fatty acid oxidation disorders: laboratory diagnosis, pathogenesis, and the complicated route to treatment. J Lipid Atheroscler. (2020) 9:313–33. doi: 10.12997/jla.2020.9.3.313, PMID: 33024728 PMC7521971

[11] Tang H Yang CZ Li H Wen W Huang FF Huang ZF. Fat emulsion tolerance in preterm infants of different gestational ages in the early stage after birth. Zhongguo Dang Dai Er Ke Za Zhi. (2017) 19:632–7. doi: 10.7499/j.issn.1008-8830.2017.06.005, PMID: 28606228 PMC7390290

[12] Greer FR. The role of pediatricians as innovators in pediatric nutrition. Nestle Nutr Workshop Ser Pediatr Program. (2010) 66:191–203. doi: 10.1159/000318958, PMID: 20664226

[13] Crill CM Helms RA. The use of carnitine in pediatric nutrition. Nutr Clin Pract. (2007) 22:204–13. doi: 10.1177/0115426507022002204, PMID: 17374794

[14] Pfanner N Warscheid B Wiedemann N. Mitochondrial proteins: from biogenesis to functional networks. Nat Rev Mol Cell Biol. (2019) 20:267–84. doi: 10.1038/s41580-018-0092-0, PMID: 30626975 PMC6684368

[15] Ahmed NY Knowles R Dehorter N. New insights into cholinergic neuron diversity. Front Mol Neurosci. (2019) 12:204. doi: 10.3389/fnmol.2019.00204, PMID: 31551706 PMC6736589

[16] Szutowicz A Bielarczyk H Zyśk M Dyś A Ronowska A Gul-Hinc S. Early and late Pathomechanisms in Alzheimer’s disease: from zinc to amyloid-β neurotoxicity. Neurochem Res. (2017) 42:891–904. doi: 10.1007/s11064-016-2154-z, PMID: 28039593 PMC5357490

[17] Szrok-Jurga S Czumaj A Turyn J Hebanowska A Swierczynski J Sledzinski T. The physiological and pathological role of acyl-CoA oxidation. Int J Mol Sci. (2023) 24:857. doi: 10.3390/ijms241914857, PMID: 37834305 PMC10573383

[18] Kelley DE Mandarino LJ. Fuel selection in human skeletal muscle in insulin resistance: a reexamination. Diabetes. (2000) 49:677–83. doi: 10.2337/diabetes.49.5.677, PMID: 10905472

[19] Drosatos K Schulze PC. Cardiac lipotoxicity: molecular pathways and therapeutic implications. Curr Heart Fail Rep. (2013) 10:109–21. doi: 10.1007/s11897-013-0133-0, PMID: 23508767 PMC3647019

[20] Ferreira GC McKenna MC. L-carnitine and acetyl-L-carnitine roles and neuroprotection in developing brain. Neurochem Res. (2017) 42:1661–75. doi: 10.1007/s11064-017-2288-7, PMID: 28508995 PMC5621476

[21] Johnston MV Trescher WH Ishida A Nakajima W. Neurobiology of hypoxic-ischemic injury in the developing brain. Pediatr Res. (2001) 49:735–41. doi: 10.1203/00006450-200106000-00003, PMID: 11385130

[22] Sylvester KG Kastenberg ZJ Moss RL Enns GM Cowan TM Shaw GM. Acylcarnitine profiles reflect metabolic vulnerability for necrotizing enterocolitis in newborns born premature. J Pediatr. (2017) 181:80–5.e1. doi: 10.1016/j.jpeds.2016.10.019, PMID: 27836286 PMC5538349

[23] Virmani A Binienda Z. Role of carnitine esters in brain neuropathology. Mol Asp Med. (2004) 25:533–49. doi: 10.1016/j.mam.2004.06.003, PMID: 15363640

[24] Schulpis KH Vlachos GD Antsaklis A Liapi C Stolakis V Zarros A. Modulated human maternal and premature neonatal erythrocyte membrane enzyme activities in relation to the mode of delivery: in vitro restoration with L-carnitine. Clin Chem Lab Med. (2011) 49:559–62. doi: 10.1515/CCLM.2011.059, PMID: 21323624

[25] Li JM Li LY Qin X Ning LJ Lu DL Li DL. Systemic regulation of L-carnitine in nutritional metabolism in zebrafish, *Danio rerio*. Sci Rep. (2017) 7:40815. doi: 10.1038/srep40815, PMID: 28102299 PMC5244368

[26] Auguet T Bertran L Binetti J Aguilar C Martínez S Guiu-Jurado E. Hepatocyte notch signaling deregulation related to lipid metabolism in women with obesity and nonalcoholic fatty liver. Obesity (Silver Spring). (2020) 28:1487–93. doi: 10.1002/oby.22873, PMID: 32657010

[27] Kon K Ikejima K Morinaga M Kusama H Arai K Aoyama T. L-carnitine prevents metabolic steatohepatitis in obese diabetic KK-A (y) mice. Hepatol Res. (2017) 47:E44–e54. doi: 10.1111/hepr.12720, PMID: 27062266

[28] Hussain A Cho JS Kim JS Lee YI. Protective effects of polyphenol enriched complex plants extract on metabolic dysfunctions associated with obesity and related nonalcoholic fatty liver diseases in high fat diet-induced C57BL/6 mice. Molecules. (2021) 26:302. doi: 10.3390/molecules26020302, PMID: 33435558 PMC7827276

[29] Molloy JW Barry D. The interplay between glucose and ketone bodies in neural stem cell metabolism. J Neurosci Res. (2024) 102:e25342. doi: 10.1002/jnr.25342, PMID: 38773878

[30] Steiner P. Brain fuel utilization in the developing brain. Ann Nutr Metab. (2019) 75:8–18. doi: 10.1159/000508054, PMID: 32564020

[31] Arima Y Nakagawa Y Takeo T Ishida T Yamada T Hino S. Murine neonatal ketogenesis preserves mitochondrial energetics by preventing protein hyperacetylation. Nat Metab. (2021) 3:196–210. doi: 10.1038/s42255-021-00342-6, PMID: 33619377

[32] Tsuruta H Yamahara K Yasuda-Yamahara M Kume S. Emerging pathophysiological roles of ketone bodies. Physiology (Bethesda). (2024) 39:2023. doi: 10.1152/physiol.00031.2023, PMID: 38260943

[33] Manta-Vogli PD Schulpis KH Dotsikas Y Loukas YL. The significant role of carnitine and fatty acids during pregnancy, lactation and perinatal period. Nutritional support in specific groups of pregnant women. Clin Nutr. (2020) 39:2337–46. doi: 10.1016/j.clnu.2019.10.025, PMID: 31732292

[34] Kępka A Chojnowska S Okungbowa OE Zwierz K. The role of carnitine in the perinatal period. Dev Period Med. (2014) 18:417–25. PMID: 25874778

[35] Fielding R Riede L Lugo JP Bellamine A. L-carnitine supplementation in recovery after exercise. Nutrients. (2018) 10:349. doi: 10.3390/nu10030349, PMID: 29534031 PMC5872767

[36] Meyburg J Schulze A Kohlmueller D Pöschl J Linderkamp O Hoffmann GF. Acylcarnitine profiles of preterm infants over the first four weeks of life. Pediatr Res. (2002) 52:720–3. doi: 10.1203/00006450-200211000-00018 12409519

[37] Baronio F Righi B Righetti F Bettocchi I Ortolano R Faldella G. Carnitine longitudinal pattern in preterm infants <1800 g body weight: a case-control study. Pediatr Res. (2019) 86:646–50. doi: 10.1038/s41390-019-0497-2, PMID: 31291643

[38] Protas PT Kępka A Rybi-Szuminska A Storonowicz J Klukowski M Wasilewska A. Are low birth weight children predisposed to renal loss of carnitine? J Matern Fetal Neonatal Med. (2020) 33:2612–7. doi: 10.1080/14767058.2018.1555813 30513037

[39] Frigeni M Balakrishnan B Yin X Calderon FRO Mao R Pasquali M. Functional and molecular studies in primary carnitine deficiency. Hum Mutat. (2017) 38:1684–99. doi: 10.1002/humu.23315, PMID: 28841266 PMC5665702

[40] Oey NA Ruiter JP Attié-Bitach T Ijlst L Wanders RJ Wijburg FA. Fatty acid oxidation in the human fetus: implications for fetal and adult disease. J Inherit Metab Dis. (2006) 29:71–5. doi: 10.1007/s10545-006-0199-x, PMID: 16601871

[41] Salguero Olid A Blanco Sanchez G Alonso OA. A systematic review about prophylactic L-carnitine administration in parenteral nutrition of extremely preterm infants. Farm Hosp. (2018) 42:168–73. doi: 10.7399/fh.10976, PMID: 29959842

[42] Crill CM Storm MC Christensen ML Hankins CT Bruce Jenkins M Helms RA. Carnitine supplementation in premature neonates: effect on plasma and red blood cell total carnitine concentrations, nutrition parameters and morbidity. Clin Nutr. (2006) 25:886–96. doi: 10.1016/j.clnu.2006.05.002, PMID: 16808989

[43] Helms RA Mauer EC Hay WW Jr Christensen ML Storm MC. Effect of intravenous L-carnitine on growth parameters and fat metabolism during parenteral nutrition in neonates. JPEN J Parenter Enteral Nutr. (1990) 14:448–53. doi: 10.1177/0148607190014005448, PMID: 2122016

[44] Bonner CM DeBrie KL Hug G Landrigan E Taylor BJ. Effects of parenteral L-carnitine supplementation on fat metabolism and nutrition in premature neonates. J Pediatr. (1995) 126:287–92. doi: 10.1016/s0022-3476(95)70562-7, PMID: 7844680

[45] Whitfield J Smith T Sollohub H Sweetman L Roe CR. Clinical effects of L-carnitine supplementation on apnea and growth in very low birth weight infants. Pediatrics. (2003) 111:477–82. doi: 10.1542/peds.111.3.477, PMID: 12612224

[46] Nałecz KA Nałecz MJ. Carnitine--a known compound, a novel function in neural cells. Acta Neurobiol Exp (Wars). (1996) 56:597–609. doi: 10.55782/ane-1996-1165, PMID: 8768311

[47] Coviello C Keunen K Kersbergen KJ Groenendaal F Leemans A Peels B. Effects of early nutrition and growth on brain volumes, white matter microstructure, and neurodevelopmental outcome in preterm newborns. Pediatr Res. (2018) 83:102–10. doi: 10.1038/pr.2017.227, PMID: 28915232

[48] Kidokoro H Anderson PJ Doyle LW Woodward LJ Neil JJ Inder TE. Brain injury and altered brain growth in preterm infants: predictors and prognosis. Pediatrics. (2014) 134:e444–53. doi: 10.1542/peds.2013-2336, PMID: 25070300

[49] Volpe JJ. Commentary-cerebellar underdevelopment in the very preterm infant: important and underestimated source of cognitive deficits. J Neonatal Perinatal Med. (2021) 14:451–6. doi: 10.3233/NPM-210774, PMID: 33967062 PMC8673497

[50] Lamhonwah AM Barić I Lamhonwah J Grubić M Tein I. Attention deficit/hyperactivity disorder as an associated feature in OCTN2 deficiency with novel deletion (p.T440-Y449). Clin Case Rep. (2018) 6:585–91. doi: 10.1002/ccr3.1316, PMID: 29636919 PMC5889263

[51] Belfort MB Inder TE. Human Milk and preterm infant brain development: a narrative review. Clin Ther. (2022) 44:612–21. doi: 10.1016/j.clinthera.2022.02.011, PMID: 35307209 PMC9133155

[52] Ottolini KM Andescavage N Kapse K Jacobs M Limperopoulos C. Improved brain growth and microstructural development in breast milk-fed very low birth weight premature infants. Acta Paediatr. (2020) 109:1580–7. doi: 10.1111/apa.15168, PMID: 31922288 PMC7347461

[53] Hortensius LM van Elburg RM Nijboer CH Benders M de Theije CGM. Postnatal nutrition to improve brain development in the preterm infant: a systematic review from bench to bedside. Front Physiol. (2019) 10:961. doi: 10.3389/fphys.2019.00961, PMID: 31404162 PMC6677108

[54] Jones A Thornton C. Mitochondrial dynamics in the neonatal brain - a potential target following injury? Biosci Rep. (2022) 42:696. doi: 10.1042/BSR20211696, PMID: 35319070 PMC8965818

[55] Natarajan G Pappas A Shankaran S. Outcomes in childhood following therapeutic hypothermia for neonatal hypoxic-ischemic encephalopathy (HIE). Semin Perinatol. (2016) 40:549–55. doi: 10.1053/j.semperi.2016.09.007, PMID: 27863707 PMC5370563

[56] Takenouchi T Sugiura Y Morikawa T Nakanishi T Nagahata Y Sugioka T. Therapeutic hypothermia achieves neuroprotection via a decrease in acetylcholine with a concurrent increase in carnitine in the neonatal hypoxia-ischemia. J Cereb Blood Flow Metab. (2015) 35:794–805. doi: 10.1038/jcbfm.2014.253, PMID: 25586144 PMC4420853

[57] McKenna MC Scafidi S Robertson CL. Metabolic alterations in developing brain after injury: knowns and unknowns. Neurochem Res. (2015) 40:2527–43. doi: 10.1007/s11064-015-1600-7, PMID: 26148530 PMC4961252

[58] Higgins RD Raju T Edwards AD Azzopardi DV Bose CL Clark RH. Hypothermia and other treatment options for neonatal encephalopathy: an executive summary of the Eunice Kennedy Shriver NICHD workshop. J Pediatr. (2011) 159:851–8.e1. doi: 10.1016/j.jpeds.2011.08.004, PMID: 21875719 PMC3263823

[59] Parikh P Juul SE. Neuroprotective strategies in neonatal brain injury. J Pediatr. (2018) 192:22–32. doi: 10.1016/j.jpeds.2017.08.031, PMID: 29031859

[60] Kochanek PM Jackson TC Ferguson NM Carlson SW Simon DW Brockman EC. Emerging therapies in traumatic brain injury. Semin Neurol. (2015) 35:83–100. doi: 10.1055/s-0035-1544237, PMID: 25714870 PMC4356170

[61] Misheva M Kotzamanis K Davies LC Tyrrell VJ Rodrigues PRS Benavides GA. Oxylipin metabolism is controlled by mitochondrial β-oxidation during bacterial inflammation. Nat Commun. (2022) 13:139. doi: 10.1038/s41467-021-27766-8, PMID: 35013270 PMC8748967

[62] Lee BJ Lin JS Lin YC Lin PT. Effects of L-carnitine supplementation on oxidative stress and antioxidant enzymes activities in patients with coronary artery disease: a randomized, placebo-controlled trial. Nutr J. (2014) 13:79. doi: 10.1186/1475-2891-13-79, PMID: 25092108 PMC4125592

[63] Pagano G Manfredi C Pallardó FV Lyakhovich A Tiano L Trifuoggi M. Potential roles of mitochondrial cofactors in the adjuvant mitigation of proinflammatory acute infections, as in the case of sepsis and COVID-19 pneumonia. Inflamm Res. (2021) 70:159–70. doi: 10.1007/s00011-020-01423-0, PMID: 33346851 PMC7750159

[64] Keshani M Alikiaii B Babaei Z Askari G Heidari Z Sharma M. The effects of L-carnitine supplementation on inflammation, oxidative stress, and clinical outcomes in critically ill patients with sepsis: a randomized, double-blind, controlled trial. Nutr J. (2024) 23:31. doi: 10.1186/s12937-024-00934-4, PMID: 38444016 PMC10916166

[65] Akisu M Ozmen D Baka M Habif S Yalaz M Arslanoglu S. Protective effect of dietary supplementation with L-arginine and L-carnitine on hypoxia/reoxygenation-induced necrotizing enterocolitis in young mice. Biol Neonate. (2002) 81:260–5. doi: 10.1159/000056757, PMID: 12011570

[66] Di Lorenzo M Bass J Krantis A. An intraluminal model of necrotizing enterocolitis in the developing neonatal piglet. J Pediatr Surg. (1995) 30:1138–42. doi: 10.1016/0022-3468(95)90006-3, PMID: 7472967

[67] Gollin G Stadie D Mayhew J Slater L Asmerom Y Boskovic D. Early detection of impending necrotizing enterocolitis with urinary intestinal fatty acid-binding protein. Neonatology. (2014) 106:195–200. doi: 10.1159/000362497, PMID: 25012466

[68] Sinclair TJ Ye C Chen Y Zhang D Li T Ling XB. Progressive metabolic dysfunction and nutritional variability precedes necrotizing enterocolitis. Nutrients. (2020) 12:275. doi: 10.3390/nu12051275, PMID: 32365850 PMC7281969

[69] Ozturk MA Gunes T Koklu E Erciyes A. Free carnitine levels in respiratory distress syndrome during the first week of life. Am J Perinatol. (2006) 23:445–9. doi: 10.1055/s-2006-951305, PMID: 17009198

[70] Korkmaz A Tekinalp G Coskun T Yigit S Yurdakok M. Plasma carnitine levels in preterm infants with respiratory distress syndrome. Pediatr Int. (2005) 47:49–52. doi: 10.1111/j.1442-200x.2005.01998.x, PMID: 15693866

[71] Zheng G Zheng J Hu X Zhu T. Decrease in lipid metabolic indexes in infants with neonatal respiratory distress syndrome. Exp Ther Med. (2024) 27:69. doi: 10.3892/etm.2023.12357, PMID: 38236433 PMC10792408

[72] Chang JL Gong J Rizal S Peterson AL Chang J Yao C. Upregulating carnitine palmitoyltransferase 1 attenuates hyperoxia-induced endothelial cell dysfunction and persistent lung injury. Respir Res. (2022) 23:205. doi: 10.1186/s12931-022-02135-1, PMID: 35964084 PMC9375342

[73] Ozturk MA Kardas Z Kardas F Gunes T Kurtoglu S. Effects of L-carnitine supplementation on respiratory distress syndrome development and prognosis in premature infants: a single blind randomized controlled trial. Exp Ther Med. (2016) 11:1123–7. doi: 10.3892/etm.2015.2964, PMID: 26998047 PMC4774440

[74] Ringseis R Keller J Eder K. Mechanisms underlying the anti-wasting effect of L-carnitine supplementation under pathologic conditions: evidence from experimental and clinical studies. Eur J Nutr. (2013) 52:1421–42. doi: 10.1007/s00394-013-0511-0, PMID: 23508457

[75] Montesano A Senesi P Luzi L Benedini S Terruzzi I. Potential therapeutic role of L-carnitine in skeletal muscle oxidative stress and atrophy conditions. Oxidative Med Cell Longev. (2015) 2015:646171. doi: 10.1155/2015/646171, PMID: 25838869 PMC4369953

[76] Das KC. Hyperoxia decreases glycolytic capacity, glycolytic reserve and oxidative phosphorylation in MLE-12 cells and inhibits complex I and II function, but not complex IV in isolated mouse lung mitochondria. PLoS One. (2013) 8:e73358. doi: 10.1371/journal.pone.0073358, PMID: 24023862 PMC3759456

[77] Yao H Gong J Peterson AL Lu X Zhang P Dennery PA. Fatty acid oxidation protects against Hyperoxia-induced endothelial cell apoptosis and lung injury in neonatal mice. Am J Respir Cell Mol Biol. (2019) 60:667–77. doi: 10.1165/rcmb.2018-0335OC, PMID: 30571144 PMC6543740

[78] O’Donnell J Finer NN Rich W Barshop BA Barrington KJ. Role of L-carnitine in apnea of prematurity: a randomized, controlled trial. Pediatrics. (2002) 109:622–6. doi: 10.1542/peds.109.4.622, PMID: 11927706

[79] Kumar M Kabra NS Paes B. Carnitine supplementation for preterm infants with recurrent apnea. Cochrane Database Syst Rev. (2004) 2003:Cd004497. doi: 10.1002/14651858.CD004497.pub2, PMID: 15495116 PMC8826756

[80] Horbar JD Ehrenkranz RA Badger GJ Edwards EM Morrow KA Soll RF. Weight growth velocity and postnatal growth failure in infants 501 to 1500 grams: 2000-2013. Pediatrics. (2015) 136:e84–92. doi: 10.1542/peds.2015-0129, PMID: 26101360

[81] Kępka A Szajda SD Waszkiewicz N Płudowski P Chojnowska S Rudy M. Carnitine: function, metabolism and value in hepatic failure during chronic alcohol intoxication. Postepy Hig Med Dosw (Online). (2011) 65:645–53. doi: 10.5604/17322693.962226, PMID: 22100797

[82] Czeczot H Scibior D. Role of L-carnitine in metabolism, nutrition and therapy. Postepy Hig Med Dosw (Online). (2005) 59:9–19. PMID: 15761381

[83] Che B Chen H Wang A Peng H Bu X Zhang J. Association between plasma L-carnitine and cognitive impairment in patients with acute ischemic stroke. J Alzheimer’s Dis. (2022) 86:259–70. doi: 10.3233/JAD-215376, PMID: 35068454

[84] Tang S Xu S Lu X Gullapalli RP McKenna MC Waddell J. Neuroprotective effects of acetyl-L-carnitine on neonatal hypoxia ischemia-induced brain injury in rats. Dev Neurosci. (2016) 38:384–96. doi: 10.1159/000455041, PMID: 28226317 PMC5411262

[85] Xu S Waddell J Zhu W Shi D Marshall AD McKenna MC. In vivo longitudinal proton magnetic resonance spectroscopy on neonatal hypoxic-ischemic rat brain injury: neuroprotective effects of acetyl-L-carnitine. Magn Reson Med. (2015) 74:1530–42. doi: 10.1002/mrm.25537, PMID: 25461739 PMC4452442

[86] Reyes-Corral M Sola-Idígora N de la Puerta R Montaner J Ybot-González P. Nutraceuticals in the prevention of neonatal hypoxia-ischemia: a comprehensive review of their neuroprotective properties, mechanisms of action and future directions. Int J Mol Sci. (2021) 22:524. doi: 10.3390/ijms22052524, PMID: 33802413 PMC7959318

